# A Rare Case of Autoimmune Enteropathy Associated With Lambert-Eaton Myasthenic Syndrome and a Literature Review

**DOI:** 10.7759/cureus.91778

**Published:** 2025-09-07

**Authors:** Georgios Chlorakis, Kalliopi Foteinogiannopoulou, Ioannis Drygiannakis, Vasileios Mastorodemos, Ioannis Ε Koutroubakis

**Affiliations:** 1 Department of Gastroenterology, University General Hospital of Heraklion, Heraklion, GRC; 2 Department of Neurology, University General Hospital of Heraklion, Heraklion, GRC

**Keywords:** autoimmune enteropathy, azathioprine, lambert-eaton myasthenic syndrome, malnutrition, non-celiac enteropathy

## Abstract

Autoimmune enteropathy is a rare intestinal disease characterized by chronic diarrhea and severe malabsorption in the presence of circulating autoantibodies against enterocytes and goblet cells, typically affecting children or young adults. Lambert-Eaton myasthenic syndrome (LEMS) is also a rare autoimmune neuromuscular junction disorder characterized by muscle weakness due to antibodies against the P/Q-type voltage-gated calcium channels (VGCCs) on the presynaptic nerve terminals. We report a case of concurrent diagnoses of autoimmune enteropathy and LEMS in a 24-year-old female patient with a history of autoimmune thyroiditis and vitiligo. The diagnosis of LEMS was established by the typical electromyographic findings and positive antibodies against P/Q-type VGCCs. After malignancies were excluded, treatment with rituximab in combination with intravenous immunoglobulin led to improvement. Within two months of the LEMS diagnosis, the patient presented with severe muscle weakness, chronic diarrhea, vomiting, and weight loss. After a thorough work-up, the diagnosis of autoimmune enteropathy was made, based on severe villous atrophy with a dense inflammatory infiltrate in duodenal biopsies and the exclusion of other causes of chronic diarrhea and non-celiac enteropathy. The patient was treated successfully with total parenteral nutrition, corticosteroids, and azathioprine. To our knowledge, this is one of the first reports of autoimmune enteropathy associated with LEMS.

## Introduction

Autoimmune enteropathy is a rare gastrointestinal disorder that typically affects children. It is associated with serum autoantibodies against enterocytes and goblet cells. The most common symptoms are chronic watery diarrhea and malabsorption, leading to growth retardation and cachexia [1]. The diagnosis lies in the exclusion of other causes of chronic diarrhea in a setting of non-celiac enteropathy. Typical histopathological findings include villous atrophy and blunting and dense infiltration by cells of innate and adaptive immunity. The disease has been mostly studied in infants and children, who are the age group of highest incidence and prevalence [2]. However, autoimmune enteropathy is increasingly recognized among young adults too [3]. Lambert-Eaton myasthenic syndrome (LEMS) is a rare disorder with an updated prevalence of 2.6 per one million. It is considered the prototypical presynaptic disorder of neuromuscular transmission, with antibodies against the P/Q-type voltage-gated calcium channels (VGCCs) responsible for the clinical symptoms of LEMS. These antibodies have been detected in 85-90% of patients with LEMS and are thought to be highly specific. Although most patients (50-60%) with LEMS have a tumor, a growing number of non-tumor LEMS (NT-LEMS) cases are recognized [4]. To the best of our knowledge, there is no report associating autoimmune enteropathy with LEMS. Both diagnoses are challenging and require a high degree of clinical suspicion. Treatments differ but converge on immunosuppression [5].

## Case presentation

A 24-year-old female patient with a history of autoimmune thyroiditis and vitiligo presented to the Neurology outpatient clinic with gait dysfunction, lower extremity weakness, and autonomic dysfunction (xerostomia and xerophthalmia) for the past 11 months. On neurological examination, proximal muscle weakness was more pronounced in the lower extremities, and decreased tendon reflexes with post-exercise facilitation were found. Electromyography and especially repetitive nerve stimulation (RNS) revealed a decrement >10% at low (3Hz) frequency and an increment >100% after maximal voluntary contraction for 20s, thus raising strong suspicion for LEMS. The diagnosis was confirmed following positive serologic testing for P/Q-type VGCC antibodies. While the workup for possible malignancy was ongoing, the patient was initially treated with intravenous gamma globulin (IgG) for five days, followed by a second course one month later, with partial improvement in proximal arm and leg weakness.

She was started on amifampridine with a slowly escalating dosage. In addition, two months after diagnosis, rituximab was initiated as maintenance therapy with the patient receiving 0.5 gm on day one and day 15 intravenously. However, while the results of serology were pending, pyridostigmine was administered, but within five days it was discontinued due to diarrhea, initially attributed to the drug. In retrospect, this gastrointestinal symptom was more likely related to the underlying autoimmune enteropathy rather than a direct adverse effect of pyridostigmine. Unfortunately, diarrhea persisted and worsened and was accompanied by vomiting.

As such, two months later, hospitalization was deemed appropriate. At that time, the patient was underweight with a body mass index of 14 kg/m^2^, had postprandial vomiting, diffuse abdominal pain without tenderness, and nine to 10 watery bowel movements per 24 hours with urgency and nocturnal awakening, but without blood or mucus. She was afebrile. Stool testing for Clostridioides difficile toxins, parasites, and bacterial pathogens was repeatedly negative. Blood tests were significant for hyperchromic, macrocytic anemia, severe hypoalbuminemia, and hyperthyroidism, although on levothyroxine for thyroiditis, and deficiencies of vitamins B12, D, and folic acid. The biomarkers of inflammation were slightly elevated, and antibodies for celiac disease were negative with normal levels of serum immunoglobulins IgA, IgG, and IgM (Table 1).

**Table 1:** Blood exams at the first consultation Slight elevation of inflammatory markers, hyperchromic, macrocytic anemia with low vitamin B12 and folic acid, thyroid dysfunction, micronutrient and electrolyte insufficiency with low albumin levels, and vitamin D deficiency were observed.

Laboratory parameter	Value	Reference range
Total leukocyte count (K/μL)	6700	4.500-10.500
Hematocrit (%)/Hemoglobin (g/dL)	30.5/9.9	36-47/12-14
Mean corpuscular volume (MCV; fL)	101	82-98
Platelet count (K/μL)	239	150-450
Erythrocyte sedimentation rate (mm/h)	25	0-15
C-Reactive Protein (mg/dL)	0.7	<0.5
Vitamin B12 (pg/mL)	83	160-950
Thyroid stimulating hormone (mIU/L)	16	0.5-5
International normalized ratio (INR)	1.3	0.8-1.2
Sodium (mEq/L)/ Potassium (mEq/L)/Magnesium (mg/dL)	134/4.1/1.6	134-144/3.5-5/1.8-2.2
Total protein (g/dL)/Serum albumin (g/dL)	3.9/1.9	6-8.3/3.5-5.5
Folic acid (ng/mL)	<2.2	2.7-17
Ferritin (ng/mL)	117	30-300
Vitamin D (ng/mL)	5	25-50
Anti-tissue transglutaminase antibodies (anti-hTG) IgA (IU/mL)	4	<5
Anti-gliadin antibodies (AGA) IgA (U/mL)	11	<15
Anti-endomysium antibodies (EMA)	Negative	<1:40
Total serum immunoglobulin A (mg/dL)	263	70-400

**Figure 1:**
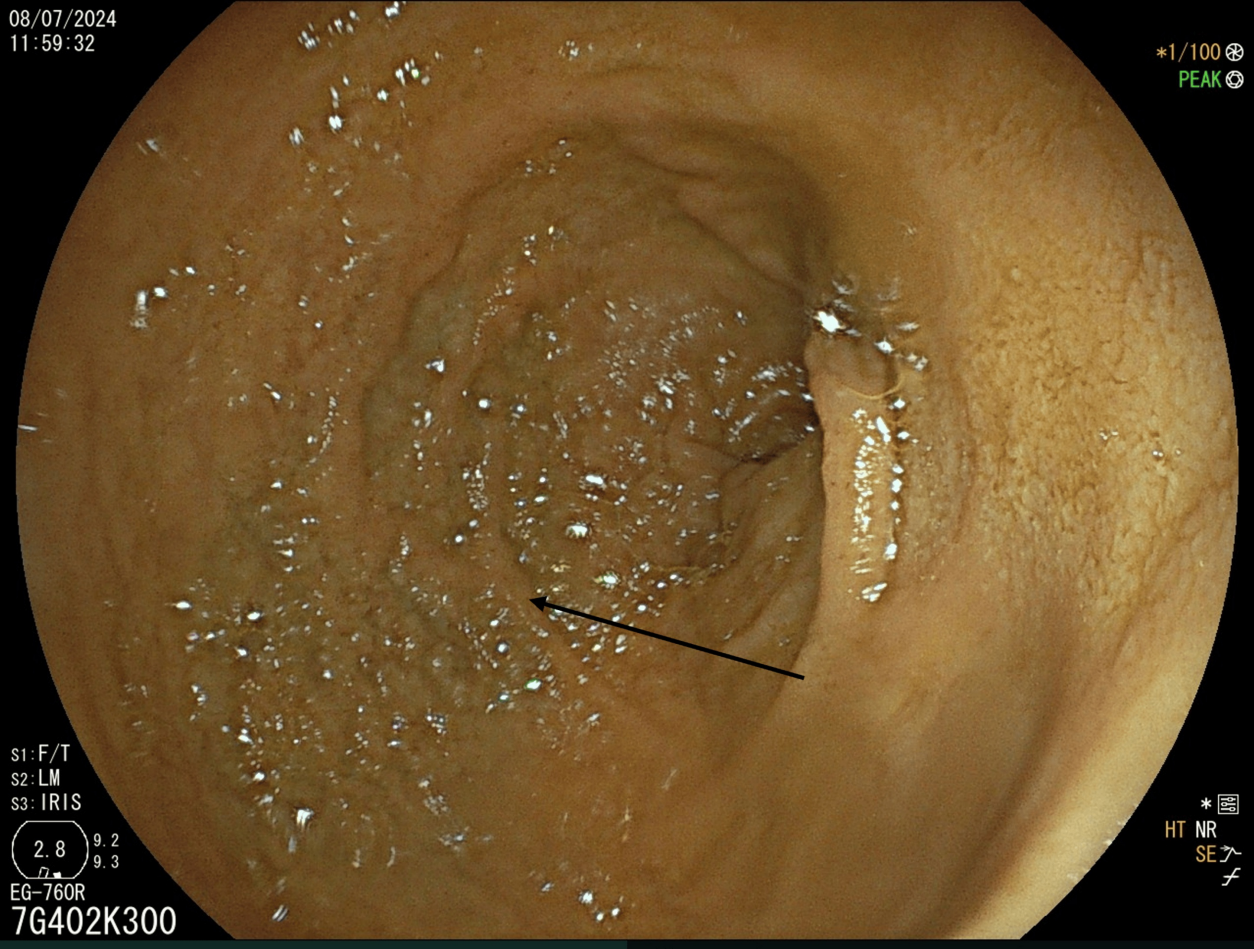
Esophagogastroduodenoscopy Slight edema (arrow) seen in the second portion of the duodenum

Esophagogastroduodenoscopy and ileocolonoscopy were performed. Edema of the duodenal mucosa was the only macroscopic finding (Figure 1). 

**Figure 2:**
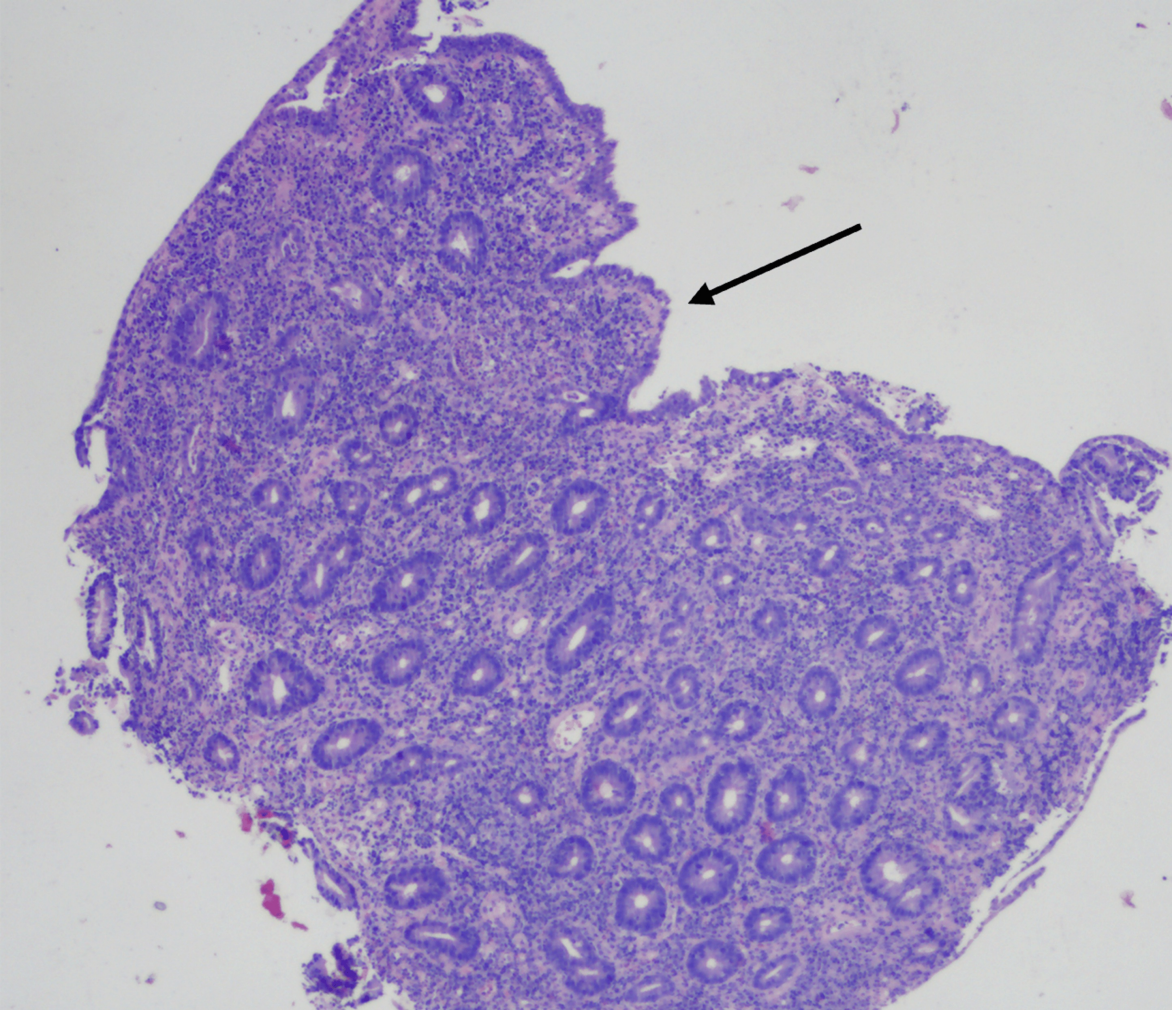
Duodenal biopsy Villous atrophy and blunting of the duodenal epithelium (hematoxylin and eosin staining).

However, microscopically, duodenal pathology was significant for marked villous atrophy and blunting. The mucosa had shortened or flattened villi, and focally there was a total absence of the glands with complete blunting (Figure 2).

**Figure 3:**
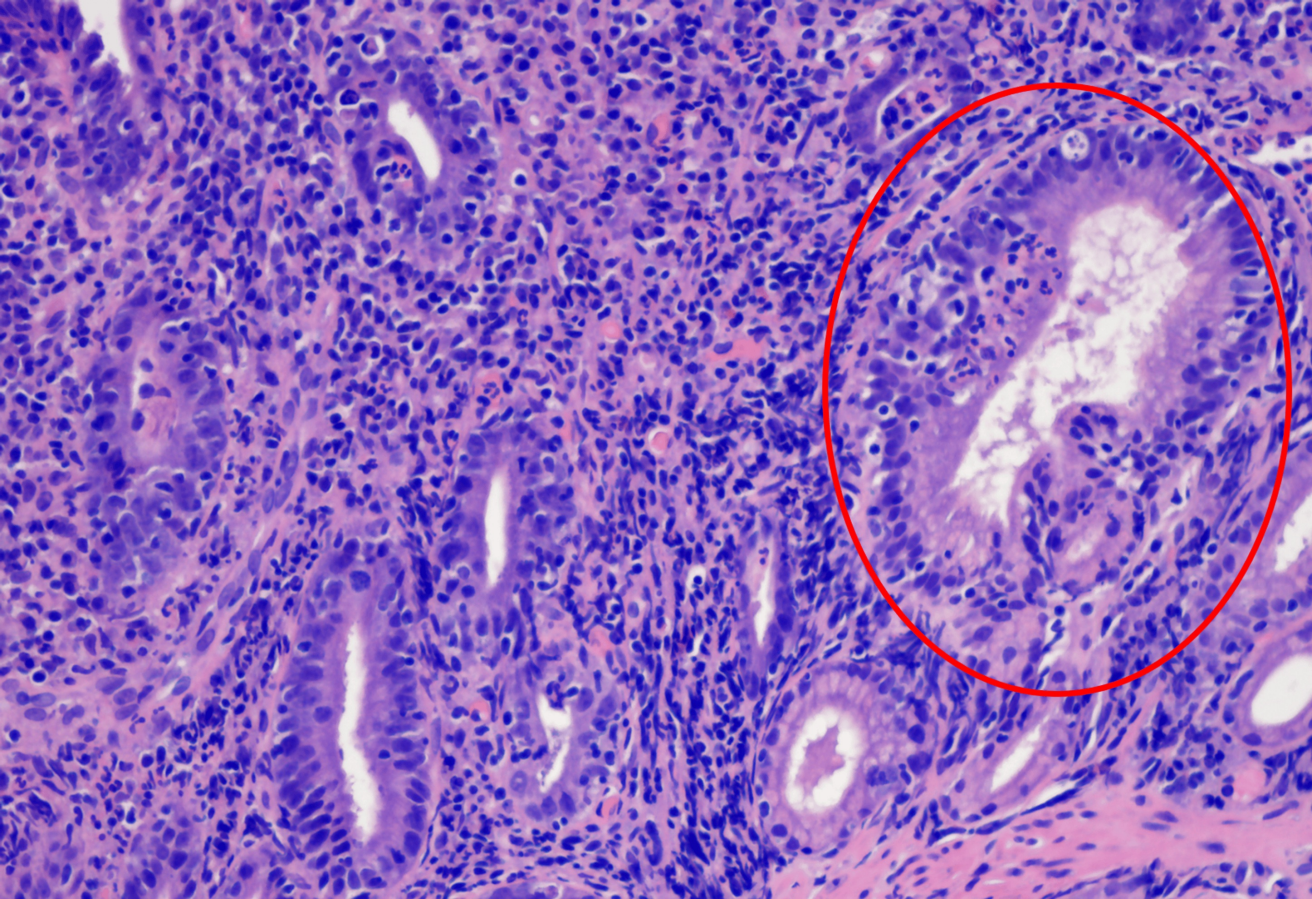
Duodenal biopsy Intraepithelial lymphocytes are less than 30 per 100 enterocytes in the duodenum (hematoxylin and eosin staining).

Same findings were reported in the stomach, in the terminal ileum, and in the colon, but with less severity. Furthermore, an expansion of the lamina propria and infiltration of the epithelial layer with cells of acute, and chronic inflammation was observed, namely, neutrophils, lymphocytes, and plasma cells. Nonetheless, the density was less than 30 intraepithelial lymphocytes per 100 epithelial cells. The infiltration was more pronounced in the lamina propria than in the deep crypts (Figure 3).

Staining for Tropheryma whipplei with the periodic acid-schiff (PAS) stain and mycobacteria with Ziehl-Neelsen stain was negative. A similar, mixed infiltrate was present in the colon and the terminal ileum. The pathologist did not identify any inclusion bodies, which would point to cytomegalovirus (CMV).

**Figure 4:**
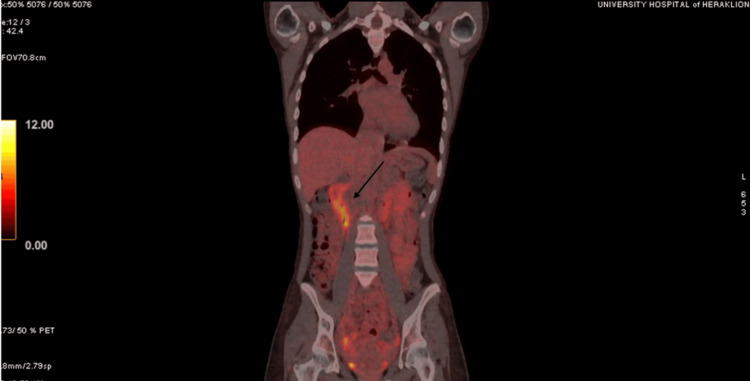
FDG-PET/CT scan FDG-PET: Fluorodeoxyglucose F 18 (18F-FDG) positron emission tomography; High metabolic activity observed in the duodenum and the small intestine (maximum standardized uptake value or SUVmax 7.26).

As LEMS most often presents as a paraneoplastic syndrome, further exploration was guaranteed. Blood gastrin, chromogranin A, and 24-hour urine 5-hydroxy-indoleacetic acid levels were normal. Computed tomography (CT) scans of the thorax and the abdomen, and CT enterography did not reveal any abnormalities in the abdominal organs or structures. Fluorodeoxyglucose F 18 (18F-FDG) positron emission tomography (PET)/CT scan showed diffusely increased metabolic activity in the duodenum and the small intestine with a maximum standardized uptake value (SUVmax) of 7.26, indicative of inflammation, rather than malignancy (Figure 4).

A diagnosis of autoimmune enteropathy was made after excluding other causes of chronic diarrhea and non-celiac enteropathy. Histology was reviewed by a senior pathologist with expertise in the gastrointestinal tract, who verified that the pathology was in line with the diagnosis of autoimmune enteropathy.

Based on the literature, and taking into account the severe clinical situation of the patient, steroids were chosen for initial treatment [6]. High doses of methylprednisolone (1 mg/kg daily, intravenous) were administered, and the patient responded with mitigation of diarrhea and cessation of vomiting. Gradually, corticosteroids were tapered, and after two weeks, azathioprine was introduced at 1 mg/kg and increased to 2 mg/kg after another week. A disease flare during methylprednisolone tapering was successfully treated by again increasing the dose and slower tapering. Because of severe malnutrition and prolonged administration of steroids at high doses, the patient had an osteoporotic fracture of the sacrum, which was treated symptomatically. Cholecalciferol was added to her medication. The patient has remained stable ever since on azathioprine, without disease recurrence and with normal blood tests.

## Discussion

A literature search in the PubMed and Google Scholar databases for full-text articles published from January 1, 2015, until July 24, 2025, was performed. The search strategy contained the terms "Autoimmune enteropathy" [Supplementary Concept]) AND "Adult"[Mesh] NOT "Celiac Disease"[Mesh] for PubMed and "autoimmune enteropathy, adults" for Google Scholar. After removing duplicates, a total of 53 articles were identified. Drug-induced cases or other causes of autoimmune enteropathy were excluded, and only primary disorders were included. Finally, 147 patients with autoimmune enteropathy were identified. The characteristics of the studies with more than three cases are summarized in Table 2.

**Table 2:** Characteristics of the studies including more than three patients with autoimmune enteropathy IMM: Immunomodulators; BA: Biologic agents

Author	Year	Patients (N)	Age (Years, Median)	Concomitant autoimmune diseases	Treatment
Charbit-Henrion et al. [7]	2023	48	22.5	52%	Systemic steroids 71%, budesonide 56%, IMM 27%, BA 54%
Villanacci et al. [8]	2019	40	20.6 (Mean)	30%	Not reported
Sharma et al. [9]	2018	30	44 (Mean)	45%	Systemic steroids 97%, IMM 30%, budesonide 30%
Li et al. [10]	2024	16	45.5	Not reported	Systemic steroids 100%, tacrolimus 43%, azathioprine 18%, thalidomide 12.5%, sirolimus 6.25%, budesonide 6.25%
van Wanrooij et al. [11]	2021	13	52	58%	Systemic steroids 61.5%, budesonide 38.5, BA 84.6%

Concomitant autoimmune diseases are common in autoimmune enteropathy: 53.8% in the cohort by van Wanrooij et al., 52% in the study by Charbit-Henrion et al., and 30% in Villanaci et al., with hypothyroidism being the most frequent [7,8,11]. Chen et al. also described associations with syndromes such as immunodysregulation polyendocrinopathy enteropathy X-linked (IPEX) syndrome and autoimmune polyglandular syndrome type 1 (APS-1), which appear more commonly in childhood [12]. Our patient had four autoimmune disorders, autoimmune thyroiditis, vitiligo, autoimmune enteropathy, and LEMS, with the latter two manifesting almost simultaneously. To the best of our knowledge, this is the first reported case of autoimmune enteropathy associated with LEMS. While autoimmune enteropathy is often linked to multiple autoimmune disorders, suggesting systemic autoimmune dysregulation, in this case the gut-brain axis appeared particularly affected.

In addition, this case expands the current understanding of autoimmune enteropathy by highlighting its potential overlap with neuromuscular autoimmune disorders. The coexistence of autoimmune enteropathy and LEMS raises the possibility of shared pathogenic mechanisms, particularly autoantibody-mediated processes. LEMS is strongly associated with antibodies targeting P/Q-type VGCCs, whereas in autoimmune enteropathy, anti-enterocyte and anti-goblet cell antibodies have been described, although inconsistently. While these antibody profiles are distinct, both conditions reflect a breakdown in self-tolerance and dysregulated B- and T-cell responses. The simultaneous onset of enteropathy and LEMS in our patient suggests that systemic immune dysregulation could extend across the gut-neuromuscular axis. Further studies are needed to clarify whether cross-reactivity or shared immune pathways contribute to this rare association.

Diagnosing the myasthenic syndrome was challenging, as LEMS is most often a paraneoplastic manifestation, commonly associated with thymoma [13]. After malignancies were excluded, LEMS was considered a primary autoimmune disorder. Regarding autoimmune enteropathy, the peculiarity of this case lies in its adult onset, as most cases are diagnosed in childhood [14]. Charbit-Henrion et al. showed that several monogenic mutations, including nuclear factor kappa B subunit 1 (NFKB1), lipopolysaccharide-responsive beige-like anchor protein (LRBA) deficiency, and cytotoxic T-lymphocyte-associated protein 4 (CTLA4) haploinsufficiency, are associated with adult onset disease [7].

Several diagnostic criteria for autoimmune enteropathy have been proposed, including the presence of anti-enterocyte antibodies [15]. However, their clinical relevance remains uncertain. They may also be positive in Crohn’s disease, celiac disease, and enteropathy-associated T-cell lymphoma (EATL) [16]. In the cohort study by Li et al., all patients tested were negative for anti-enterocyte and anti-goblet cells antibodies [10]. Thus, while these antibodies may support the diagnosis, they are not required [16]. Autoimmune enteropathy is primarily a diagnosis of exclusion, and clinicians should maintain a high index of suspicion in cases of non-celiac enteropathy with villous atrophy. In our case, villous atrophy of unexplained origin, coupled with steroid responsiveness, supported the diagnosis, even in the absence of specific antibodies or macroscopic mucosal lesions.

Endoscopic findings in autoimmune enteropathy are often nonspecific. In our patient, upper and lower GI endoscopy showed only edema of the duodenal mucosa. Previous reports have reported nodularity in 63%, erosions in 56%, and duodenal scalloping in 50% cases [10]. The discrepancy between the endoscopic findings and clinical severity is well recognized. Therefore, biopsy sampling from the stomach, duodenum, terminal ileum, and colon is recommended to delineate the extent of the disease. Capsule endoscopy may also aid in diagnosis by revealing diffuse villous atrophy [17].

Histopathology in our patient was more severe in the duodenum, although the stomach, the terminal ileum, and the colon were also involved. The findings were consistent with active chronic duodenitis (ACD), as classified by Masia et al., showing villous blunting and expansion of the lamina propria by inflammatory infiltrates. This is the most common pattern reported in the literature [18].

Treatment for autoimmune enteropathy relies on immunosuppression. Li et al. and Sharma et al. reported initial treatment with corticosteroids in all patients [9,10]. For steroid-refractory disease, immunomodulators or biologics may be required. Sharma et al. noted an 85% response rate with oral budesonide [9]. Other agents reported with success include thalidomide, sirolimus, rituximab, infliximab, adalimumab, cyclosporine, and mycophenolate mofetil [6]. In our patient, rituximab did not improve the intestinal symptoms. Intravenous immunoglobulin has been effective in some thymoma-associated cases, but in our patient, it improved only the muscle weakness [19]. Ultimately, corticosteroids combined with azathioprine induced remission, which has been maintained with azathioprine monotherapy. Notably, this regimen is effective for both autoimmune enteropathy and LEMS.

This study has several limitations. First, the anti-enterocytes and anti-goblet cells were not tested, though their diagnostic value is limited. Second, capsule endoscopy was not performed. Third genome testing for LEMS was not undertaken, but it would not have altered the clinical management. Fourth, the single case report limits generalizability; causality between LEMS and enteropathy cannot be proven. The strength of the study lies in the literature review, which confirmed the rarity of the case. No cases with concomitant autoimmune enteropathy and LEMS were identified. The diagnosis was supported by expert pathological review and confirmation of LEMS by highly specific testing for P/Q-type VGCC antibodies. This study highlights the importance of clinical suspicion in cases of refractory diarrhea with malnutrition and villous atrophy, and highlights the frequent coexistence of multiple autoimmune conditions in autoimmune enteropathy. Our patient uniquely presented with four concomitant autoimmune disorders. The simultaneous onset of enteropathy and LEMS suggests a possible shared autoimmune mechanism. Furthermore, this case reinforces that antibodies to anti-enterocytes and anti-goblet cells are not mandatory for diagnosis.

## Conclusions

Autoimmune enteropathy associated with LEMS is an exceptionally rare and dramatic manifestation of autoimmunity. Their concurrent appearance complicates the diagnostic process, as muscle weakness may result from either the neurological disorder or severe malnutrition. Combination treatment with corticosteroids and azathioprine proved to be effective in our patient. Further research is needed to elucidate the pathophysiological link between these entities and to optimize therapeutic strategies.
